# Role of Polymer Architecture in CO_2_ Capture from Air Using Supported Poly(alkylenimine)s: Linear vs Branched Polymers

**DOI:** 10.1021/acsapm.5c03465

**Published:** 2025-11-17

**Authors:** Jacob Hoffman, Laura Proaño, Christopher W. Jones

**Affiliations:** School of Chemical & Biomolecular Engineering, Georgia Institute of Technology, 311 Ferst Dr., Atlanta, Georgia 30332, United States

**Keywords:** direct air capture, adsorbents, stability, amine efficiency, cyclic capacity, desorption energy

## Abstract

Direct air capture (DAC) of CO_2_ coupled with geologic storage is a promising climate change mitigation strategy, with some applications employing amines supported on porous solids as CO_2_ sorbents. While branched poly­(ethylenimine) (PEI) is the standard benchmark amine material, it suffers from limited oxidative stability. Poly­(propylenimine) (PPI), as an alternative, has previously demonstrated improved resistance to degradation under harsh oxidative conditions. Linear and branched PEI are commercially available, though at different molecular weights, while PPI is not commercially available. For this reason, a comparative study of all four polymers (linear PEI, branched PEI, linear PPI, branched PPI) has not been reported for DAC. In this study, we synthesize and compare low-molecular-weight (∼800 g/mol) linear (L) and branched (B) PEI and PPI supported on a model support, SBA-15 silica. These materials are evaluated for CO_2_ adsorption under dry, DAC-relevant conditions (400 ppm of CO_2_, 30 °C). LPPI exhibited the highest amine efficiency at all loadings, reaching a maximum of 0.14 mmol CO_2_/mmol N, outperforming BPEI, while LPEI consistently showed the lowest uptake capacity. Temperature-programmed desorption reveals that the structure of the amine polymer impacts the CO_2_ binding strength, with branched polymers displaying higher desorption energies of 102–111 kJ/mol. *In situ* infrared spectroscopy experiments show that all sorbents preferentially capture CO_2_ as ammonium carbamate. Isobaric CO_2_ uptake studies further underscore the influence of polymer mobility and support pore crowding on performance, while demonstrating the sorbents’ performance at elevated temperatures and CO_2_ concentrations. All materials demonstrated good stability over 25 adsorption–desorption cycles using thermal regeneration in an inert gas purge, with only BPPI displaying a 10–11% decrease in capacity/amine efficiency during cycling, possibly due to the loss of low molecular weight, oligomeric amines. This is the first side-by-side comparison of the CO_2_ sorption properties of linear and branched PEI and PPI with similar molecular weights. These findings highlight the significant role of polymer architecture in CO_2_ capture efficiency and inform future designs of durable, high-performance DAC sorbents.

## Graphical abstract

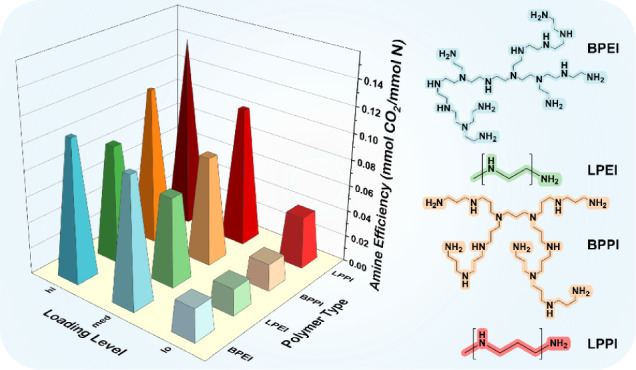

## Introduction

The increasing concentration of carbon dioxide (CO_2_) in the atmosphere since rapid industrialization and the initial usage of fossil fuels in the 19th century is widely regarded as the primary cause of climate change.^1^ The speedy and widespread rollout of carbon-neutral energy sources, combined with the retirement of carbon-intensive ones, is and will continue to be the most significant measure to combat rising greenhouse gas levels.^2^ However, atmospheric carbon removal technologies are gaining traction as the largest countries fall behind their emissions targets. Among these, the removal of carbon dioxide from the atmosphere by chemical means, also referred to as direct air capture (DAC) of CO_2_, is one of the most scalable.^3–5^ When combined with geologic sequestration on a large scale, DAC can provide a way to reduce the atmospheric CO_2_ concentration.^6^

The most well-studied mode of DAC is temperature swing adsorption or temperature-vacuum swing adsorption. Solid amine adsorbents are among the most effective DAC adsorbents, offering excellent CO_2_ capacities and selectivities to CO_2_ over N_2_ and O_2_.^7,8^ The prototypical DAC sorbent uses low molecular weight (∼800 Da), branched poly­(ethylenimine) (BPEI) supported on a porous support such as silica, alumina, zeolites, MOFs or COFs.^9–16^ BPEI is highly effective and its high primary and secondary amine density, existence as a viscous liquid with low volatility, and its commercial availability make it a popular ingredient in DAC sorbents. However, it suffers from low stability in oxidative environments, and this limits its useful lifetime, leading to high sorbent replacement costs, currently limiting its large-scale use.^17–23^

For these reasons, the development of alternative supported amines based on impregnated polymers or grafted aminosilanes has been a focus of research for over a decade.^16,24–26^ Among the oligomeric or polymeric amines, both main chain aminopolymers such as poly­(propylenimine) and side chain aminopolymers such as poly­(allylamine) and poly­(glycidylamine) have been developed.^27–30^ In a prior study, small, molecular analogues of poly­(ethylenimine) and poly­(propylenimine) were supported on a porous silica support and evaluated for CO_2_ capture under simulated DAC conditions.^31^ The propylenimine structures offered competitive CO_2_ sorption capacities and enhanced stability toward oxidation compared to the ethylenimine analogues.^31,32^ Subsequent studies of linear poly­(propylenimine) (LPPI) and branched poly­(propylenimine) (BPPI) at several molecular weights demonstrated these polymers to be potential alternatives to the BPEI materials used ubiquitously around the world as an amine DAC benchmark material.^33–35^ However, both LPPI and BPPI are not widely commercially available. Furthermore, the linear poly­(ethylenimine) analogue, LPEI, while commercially available, is only available at higher molecular weights, with the lowest molecular weight commonly offered 2500 DA. This makes direct comparisons to commercial BPEI challenging.^36–39^

In this work, we prepare LPEI, LPPI and BPPI in the laboratory at a common molecular weight to enable a direct, side-by-side comparison of the effectiveness of these different amine polymers to commercial BPEI under simulated, dry DAC conditions. Because the benchmark BPEI is ∼ 800 DA molecular weight, oligomeric LPEI, LPPI and BPPI were made at similar molecular weights, characterized, and then impregnated into a mesoporous silica SBA-15 model support material. The results demonstrate that LPPI slightly outperforms BPEI regarding dry CO_2_ capacity at 400 ppm of CO_2_ conditions, though all of the amine polymers offer useful CO_2_ sorption performance at 30 °C. To our knowledge, this is the first side-by-side comparison of these four polymers at comparable conditions, on a common support, for DAC.

## Experimental Section

### Materials

Methanol, hydrobromic acid (48%), ethylenediamine, 1,3 diaminopropane, 2-ethyl-2-oxazoline, triethylamine, Ambersep 900­(OH), Pluronic *p*-123 block copolymer and tetraethyl orthosilicate were acquired from Sigma-Aldrich. 3-amino-1-propanol and anhydrous zinc chloride were acquired from Alfa Aesar. Anhydrous acetonitrile, calcium hydride and azetidine (98%) were acquired from Thermo Scientific. Methyl-*p*-toluenesulfonate was acquired from TCI America. Ammonium hydroxide was acquired from Fisher Scientific. Hydrochloric acid (37%) was acquired from VWR. All gases were sourced from Airgas.

## Methods

### Synthesis of SBA-15

The synthesis procedure for SBA-15 followed previously published methods.^33^ In a typical synthesis, 27.6 g of Pluronic *p*-123 coblock polymer was added to a 2 L conical flask. Next, 732 g of deionized water was added, followed by 130 mL of 37% hydrochloric acid and the contents were stirred for 3 h at room temperature until solids were fully dissolved. Then, 50.1 g of tetraethyl orthosilicate was added dropwise and the contents stirred for 20 h at 40 °C. The flask was capped, insulated and kept undisturbed in an oil bath at 120 °C for 24 h. The mixture was cooled and 450 mL of deionized water was added to quench the reaction. The precipitate was washed with deionized water during vacuum filtration several times until the filtrate was clear. The precipitate was transferred to a ceramic dish and dried for 12 h at 75 °C. The solid was then calcined using the following procedure: ramp to 200 °C at 1.2 °C per min; hold for 1 h; ramp to 550 °C at 1.2 °C per min; hold for 12 h; cool to room temperature. The powder was finally collected and stored under ambient conditions.

### Linear Poly­(propylenimine) Synthesis

The synthesis of linear poly­(propylenimine) was adapted from Pang et al.^33^ Anhydrous acetonitrile (115 mL, 2.2 mol) was added to a 500 mL round-bottom flask along with a stir bar. ZnCl_2_ (5.5 g, 0.04 mol) was added all at once and dispersed. 3-amino-1-propanol (150.2 g, 2 mol) was added dropwise while stirring. The flask was heated to 95 °C and held for 48 h under refluxing. The mixture was cooled to room temperature and excess acetonitrile was removed by rotary evaporation at 30 °C. The mixture was vacuum distilled to isolate the 6-member ring monomer 2-methyl-5,6-dihydro-4H-1,3-oxazine (MeOZI).

MeOZI and acetonitrile were dried by stirring each individually with calcium hydride overnight. They were collected by vacuum distillation in respective Strauss flasks, which were backfilled with argon until proceeding in subsequent steps. Dried MeOZI (10.3 g, 0.1 mol) was added via needle to a torch-dried and argon-purged Schlenk tube capped with a rubber septum and with a stir bar inside. Dried acetonitrile (52 mL, 1 mol) was added via needle and the mixture was stirred briefly. With a strong argon flow applied through the neck of the tube, methyl-p-toluenesulfonate (2.16 g, 0.0116 mol) was added by briefly removing the septum. The mixture was purged by bubbling argon for 1 h. It was then submerged in an oil bath and left stirring for 48 h at 90 °C. 1,3-diaminopropane (1.95 mL, 0.025 mol) was added via needle and the reaction was cooled to room temperature. Acetonitrile and 1,3-diaminopropane were removed by rotary evaporation at 50 °C.

Hydrolysis of the side chain was performed by stirring with 5 M HCl (200 mL) for 48 h at 100 °C under reflux. The flask was cooled, and acetic acid was removed via rotary evaporation at 50 °C. The resulting slurry was stirred in an ice bath, 10 M NaOH was added dropwise to adjust the pH to 13 and then centrifuged. The precipitate was washed twice with NH_4_OH (14.8M), once with a 1:4 mixture of triethylamine in DI water and once with only DI water, centrifuged after each wash. The final precipitate was dissolved in methanol, filtered and dried via rotary evaporation and high vacuum (<20 mTorr). ^1^H NMR (500 MHz, CD_3_OD) was performed to ascertain the molecular weight of the polymer (Figure S6).

### Linear Poly­(ethylenimine) Synthesis

The synthesis of linear PEI was adapted from previously detailed procedures.^33,40^ Anhydrous acetonitrile and 2-ethyl-2-oxazoline were dried by stirring each individually with calcium hydride overnight. The two were isolated by vacuum distillation, collecting each in respective Strauss flasks, which were backfilled with argon until the next step. Dried 2-ethyl-2-oxazoline (8.14 g, 0.08 mol) was added via needle to a torch-dried and argon-purged Schlenk tube capped with a rubber septum with a stir bar inside. Dried acetonitrile (62 mL, 1.19 mol) was added via needle and the mixture was stirred briefly. With a strong argon flow applied through the neck of the tube, methyl-p-toluenesulfonate (1.28 g, 0.0067 mol) was added by briefly removing the septum. The mixture was purged by bubbling argon for 1 h. It was then submerged in an oil bath and left stirring for 48 h at 80 °C. Ethylenediamine (0.9 mL, 0.014 mol) was added via needle and the reaction was cooled to room temperature. Acetonitrile and ethylenediamine were removed by rotary evaporation at 50 °C.

Hydrolysis of the side chain was performed by stirring with 5 M HCl (162 mL) for 48 h at 110 °C under reflux. The flask was cooled and propanoic acid was removed via rotary evaporation at 50 °C. The resulting slurry was stirred in an ice bath, 10 M NaOH was added dropwise to adjust the pH to 13 and then centrifuged. The precipitate was washed twice with NH_4_OH (14.8M) and once with DI water, centrifuged after each wash. The final precipitate was dissolved in methanol, filtered and dried via rotary evaporation and high vacuum (<20 mTorr). ^1^H NMR (500 MHz, CD_3_OD) was performed to attain the molecular weight of the polymer (Figure S7).

### Branched Poly­(propylenimine) Synthesis

The synthesis of branched PPI was adapted from the method developed by Sarazen et al.^34,35^ Methanol (20 mL, 0.49 mol) and azetidine (98%, 1.31 mL, 0.02 mol) were added to a pressure flask with a stir bar. HBr (48%, 540 μL) was added and the flask sealed. The reaction was stirred at 90 °C for 88 h then cooled to room temperature. Methanol was removed by rotary evaporation at 30 °C. The result was solubilized in 50 mL of DI water, to which 70 g of AmberSep 900­(OH) resin was added and stirred very slowly (50 rpm) for 48 h. The mixture was filtered and the liquid dried by rotary evaporation. A second resin treatment was performed by the same process with 30 mL of DI water and 50 g of resin. After drying, the polymer was solubilized in methanol, passed through a syringe filter (0.22 μm PTFE) and dried by rotary evaporation and high vacuum (<20 mTorr). ^1^H NMR (500 MHz, D_2_O) was performed. Matrix-assisted laser desorption/ionization (MALDI) mass spectrometry was performed to attain the molecular weight of the polymer (Figure S5).

### Composite Preparation

Composites of polymer and SBA-15 were prepared via a wet impregnation method. The desired amount of polymer was dissolved in 5 mL of methanol. Meanwhile, the desired amount of SBA-15 and 15 mL of methanol were added to a 100 mL round-bottom flask and left to stir for at least 1 h to uniformly disperse. The polymer solution was transferred to the support suspension and left to stir overnight. The methanol was removed by rotary evaporation at 50 °C then dried overnight at room temperature under high vacuum (<20 mTorr). The dried composites were collected and stored under argon in wrapped glass vials.

### Organic Combustion to Assess Polymer Loading

Organic combustion experiments were performed using a TA Instruments TGA 550 to measure the organic content of the sorbents (Figure S4). Samples were held at 110 °C under a nitrogen purge for 1 h to desorb water and other adsorbed species. The purge gas was switched to air and the temperature was ramped 10 °C/min to 700 °C and held there for an additional 30 min. The mass loss between 110 °C and the end of the experiment was normalized by the residual mass to obtain the organic content.

### Nitrogen Physisorption

Micromeritics Tristar II Plus was used to perform nitrogen physisorption at 77 K. Samples were dried at 60 °C on a vacuum line reading 30 Torr for 12 h. Surface area was estimated by applying BET theory between P/P_0_ 0.05–0.2. Total pore volume was determined using the amount adsorbed at P/P_0_ of 0.95. Pore filling was calculated by the difference in total pore volume normalized by the pore volume of the blank support.

### CO_2_ Adsorption

The pseudoequilibrium CO_2_ sorption capacities of the sorbent were determined using a TA Instruments TGA 550. The powdered sorbents (∼10 mg) were held at 110 °C for 3 h under a nitrogen stream. The temperature was ramped down to the adsorption temperature of 30 °C, the inlet gas switched to 400 ppm of CO_2_ in nitrogen and held for 12 h. The samples were desorbed by ramping to and holding for 1 h at 110 °C under nitrogen.

### Temperature-Programmed Desorption

Temperature-programmed desorption was performed following 12 h of adsorption under 400 ppm of CO_2_ at 30 °C in a TGA. The samples (∼10 mg) were held for 1 h under a nitrogen purge at the adsorption temperature. The temperature was then ramped to 110 °C at 0.5 °C/min and held at 110 °C for 1 h. The experiment was repeated with fresh samples using ramp rates of 0.3 and 1.0 °C/min. A LiCOR 850 CO_2_/H_2_O analyzer was utilized to monitor the CO_2_ concentration of the outlet through the entire process.

### CO_2_ Isobars

CO_2_ isobars were measured using a TA Instruments TGA Q500. Samples (∼8 mg) were activated for 390 min at 110 °C under N_2_, after which they were cooled and equilibrated at 30 °C. The gas flow was switched to 400 ppm or 10% CO_2_ in N_2_ for 390 min. The temperature was then raised at 10 °C/min to 40 °C and held for 390 min. This was repeated in 10 °C increments up to 90 °C and then reversed back down to 30 °C, holding for 390 min at each temperature.

### Thermal Sorption–Desorption Cycling

A TA Instruments Q500 TGA was used. Samples (∼8 mg) were kept at 110 °C for 3 h under a nitrogen stream. The temperature was lowered at 10 °C/min to 30 °C and the flow switched to 400 ppm of CO_2_/N_2_ for 1 h of adsorption. The flow was then reverted to nitrogen and the temperature ramped at 10 °C/min to 110 °C, stabilized and kept for 10 min for desorption. This adsorption–desorption cycling was repeated 24 more times.

### Diffuse Reflectance Infrared Fourier Transform Spectroscopy

*In situ* FT-IR spectroscopy was performed on an Invenio Bruker IR spectrometer with a low-temperature diffuse reflectance infrared Fourier transform spectroscopy (DRIFTS) cell (CHC–CHA-4, Harrick Scientific Products Inc.). The sample holder inside the DRIFTS cell chamber was filled with about 15–20 mg of sample. The sample was activated at 100 °C under 70 mL/min Ar flow for 1 h. The temperature was cooled to 30 °C and the inlet gas flow was switched to dry 400 ppm of CO_2_/N_2_ (70 mL/min). During the adsorption, 400 spectra were collected with 60 scans at a resolution of 4 cm^–1^ every 30 s for a total period of ∼ 200 min.

## Results and Discussion

### Sorbent Characteristics

Linear poly­(ethylenimine) (*M*_n_ ≈ 740 g/mol), linear poly­(propylenimine) (*M*_n_ ≈ 760 g/mol) and branched poly­(propylenimine) (*M*_n_ ≈ 740 g/mol) were synthesized according to the methods described in the Experimental Section. Physically impregnated composites of aminopolymers in SBA-15 were prepared by a wet impregnation method, targeting low (4.4 ± 0.5 mmol N/g SBA-15), medium (10.7 ± 1.3 mmol N/g SBA-15) and high (17.8 ± 1.9 mmol N/g SBA-15) amine loadings. Organic and amine loadings were verified by combustion TGA and the BET surface areas and pore volumes were obtained from cryogenic N_2_ physisorption data (Table 1). As expected, surface area and pore volume decreased with increasing polymer loading due to the deposition of amine polymer within the pores. Of all the composite types, LPEI-impregnated SBA-15 appeared to retain significantly more of its pore volume, with only 68% of pore volume occupied even at the highest polymer loading. Compared to the others, which all show 90–100% of their pore volume occupied at the highest amine loading, this is remarkably low and suggests that the packing of LPEI is particularly effective, as revealed by previous studies, and/or more LPEI deposits on the silica’s external surface.^41–43^ Meanwhile, BPPI appears to occupy more space within the pores than the others at all loading levels on a per amine basis, likely a result of its branched architecture and the additional −CH_2_– in its structure compared to PEI (Table S2).

**Table 1:** Physical Characteristics of Aminopolymer-Impregnated SBA-15 Sorbents

	Organic loading [wt %]	Amine loading [mmol N/g SBA-15]	B.E.T. Surface Area [m^2^/g]	Pore volume [cm^3^/g]	Pore fill [%]
SBA-15	---	---	790	0.93	---
BPEI low	17	4.9	407	0.65	30
BPEI med	36	13.0	148	0.26	72
BPEI high	46	19.5	44	0.08	91
LPEI low	15	4.1	467	0.72	23
LPEI med	29	9.8	288	0.49	48
LPEI high	42	17.1	179	0.30	68
BPPI low	18	3.9	374	0.59	36
BPPI med	37	10.3	143	0.26	73
BPPI high	48	15.9	31	0.05	95
LPPI low	21	4.9	424	0.69	27
LPPI med	35	9.6	211	0.37	60
LPPI high	51	18.7	12	0.03	97

### CO_2_ Adsorption

CO_2_ capacities were measured using 400 ppm of CO_2_ in nitrogen to attain the capacity of the four sorbent types at all amine loadings (Figure 1). The sorbents were activated at 110 °C for 3 h, followed by adsorption at 30 °C for 12 h. All materials display an increase in capacity with higher amine loading, as expected. Linear PEI displays the lowest capacity and amine efficiency at all polymer loadings. Linear PEI-based sorbents have previously been observed to underperform compared to BPEI when exposed to pure CO_2_, which was attributed to LPEI having a lower mobility within the support pores.^44^ Linear PEI is a semicrystalline solid and branched PEI is viscous liquid in the bulk; it has been suggested that that remains true within the pores, accounting for the difference.^44^ Additionally, branching within BPEI affords it several primary amine sites per molecule, whereas LPEI, as synthesized, only has primary amines at the chain ends, the rest being secondary amines. Primary amines have been previously shown to be more effective at capturing CO_2_, particularly at ultradilute concentration, leading to the superior performance of BPEI.^45,46^

**Figure 1:**
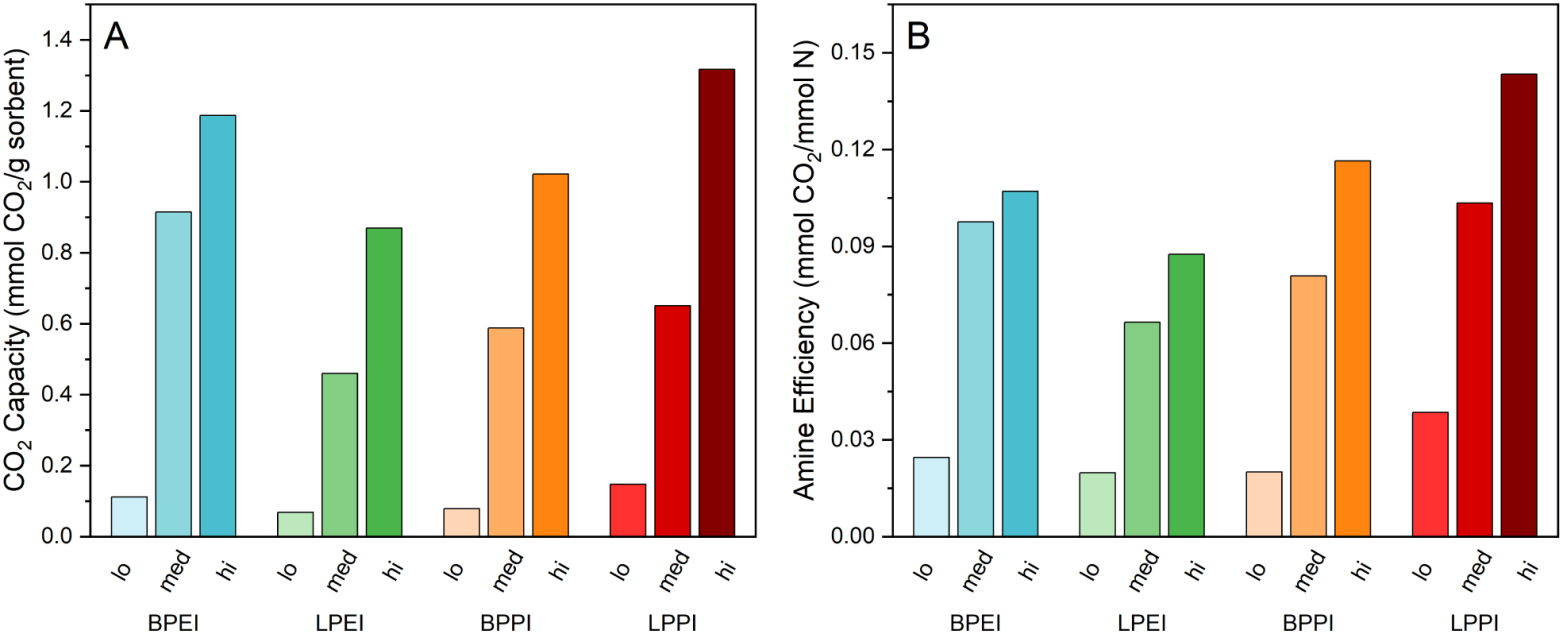
(A) CO_2_ capacity and (B) amine efficiencies of all four polymers in SBA-15 at each loading level. Adsorption performed at 30 °C and 400 ppm of CO_2_/N_2_ balance for 12 h following 3 h activation at 110 °C.

In terms of capacity, the BPPI-impregnated SBA-15 samples displayed lower uptake than the corresponding BPEI composites across all loading levels. On a weight basis, as with CO_2_ capacity, BPPI modestly underperforms BPEI due to the additional carbon in the polymer chains. On an amine basis, though, its performance is closer to that of BPEI, which could be due to the larger intramolecular distance between amine sites. The cooperation of two amine sites is crucial to the formation of alkylammonium carbamate, the adsorbed form of CO_2_, and the increased distance between these sites due to the propyl spacer can isolate them such that this coordination is more difficult. Only at the highest organic loading does BPPI have a slightly higher amine efficiency than BPEI (0.12 vs 0.11 mmol CO_2_/mmol N, respectively) which could be due to the higher amine density of BPEI or a higher degree of intermolecular amine coordination that only becomes a factor with overcrowding of the pores.

While BPEI has the highest capacity (0.91 mmol/g) of all the medium loading samples, LPPI overtakes it in terms of amine efficiency due to the higher amine density of PEI compared to PPI. In fact, linear PPI sorbents display the highest amine efficiency at all loading levels (Table S1). This could be due to the increased basicity of amine sites between propyl spacers compared to those separated by ethyl spacers.^47^ Additionally, linear PPI in the bulk is a partially crystalline solid powder, whereas branched PEI is a viscous liquid (Figure S9). This suggests that linear PPI chains are capable of cross chain coordination that aligns amine groups, thereby promoting ammonium carbamate formation. This might imply that linear PEI, which is also a solid in the bulk, should benefit from the same arrangement, though the results here show it has the worst performance. However, the fractional pore fillings for the linear PEI samples are the lowest of all materials (Table 1). The highest loaded LPEI sorbent has a pore filling of only 68%, on par with the medium loading of the other three composites. This would suggest that cross-chain coordination and hydrogen bonding is so effective that LPEI forms a tightly packed polymer layer along the pore walls and the CO_2_ may struggle to penetrate it at the low DAC CO_2_ concentration, or that there is excessive LPEI deposited on the silica external surface, with that dense film also making some amine sites inaccessible. However, XPS results suggest that the surface of LPEI-impregnated SBA-15 did not have a disproportionate concentration of polymer, probed via the nitrogen concentration, compared to the general polymer concentration of the samples, as measured by combustion TGA (Table S3).

### CO_2_ Desorption

Temperature-programmed desorption (TPD) was performed to study the binding strength of CO_2_ on the materials following 12 h of adsorption using 400 ppm of CO_2_. Initially, the temperature was held at 30 °C for 1 h under a nitrogen stream to purge physisorbed species,^48^ and then the temperature was ramped at 0.5 °C/min to 110 °C while monitoring the outlet CO_2_ concentration. The temperature at which the concentration peaked is referred to as T_peak_ and is labeled for each amine species in Figure 2A. The location of this peak is indicative of a combination of the strength of the chemisorption and the polymer properties, such as polymer chain mobility. Other factors, like support pore and particle size, can be removed from consideration, since the same parent support batch was used in all materials.

**Figure 2:**
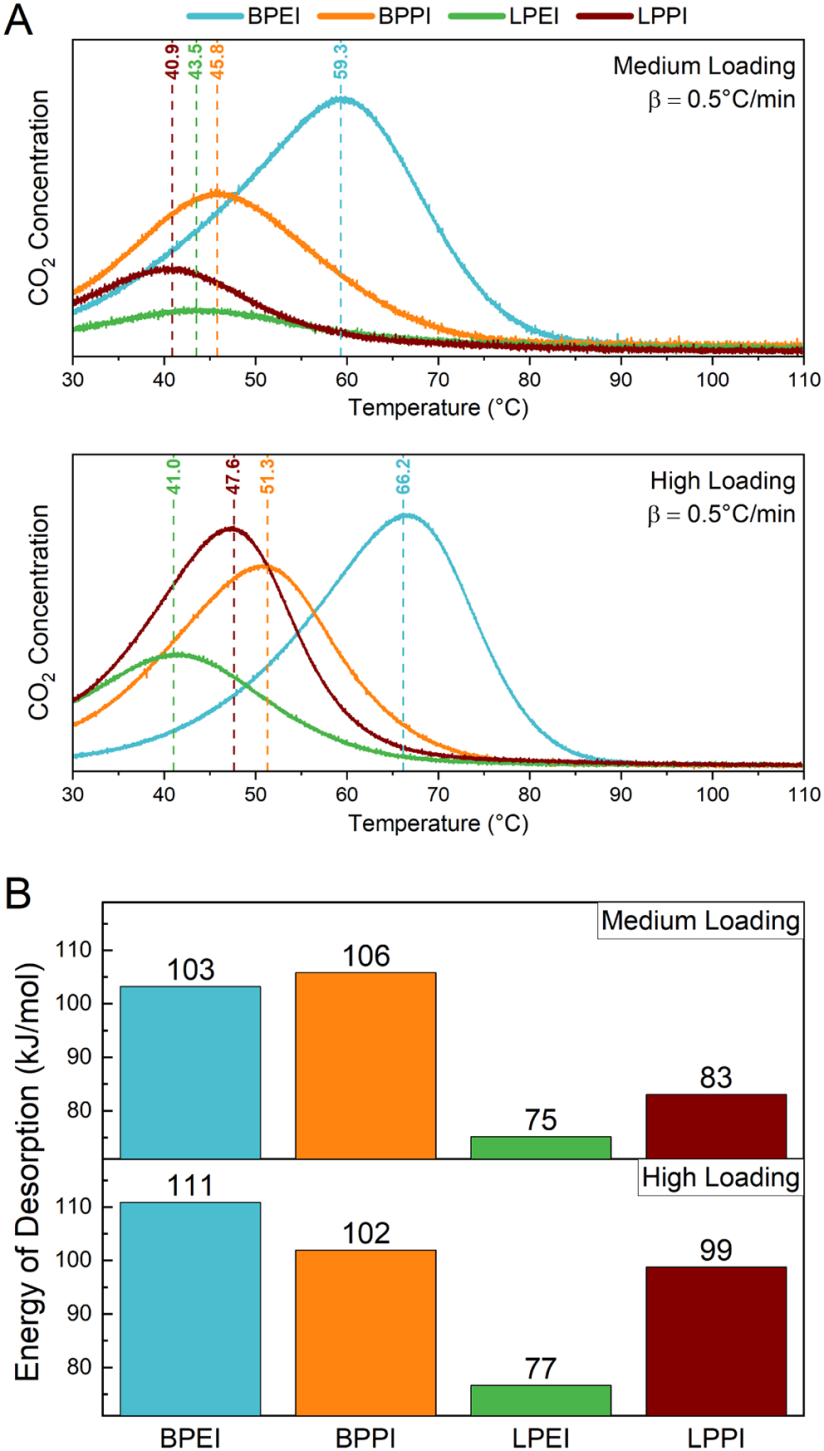
A) CO_2_ TPD profiles and B) energy of CO_2_ desorption of medium and high loaded aminopolymer/SBA-15 composites following 12 h of adsorption under 400 ppm of CO_2_ at 30 °C. Energy of desorption (E_d_) was obtained by following the method detailed by Cvetanovic et al.^52^ β: temperature ramp rate during TPD.

The desorption profiles show that the branched polymer species display higher T_peak_ values than those of the linear species, with BPEI having a significantly higher T_peak_. This can be due in part to the preferred formation of ammonium carbamate, a stronger chemisorption product than carbamic acid, on his material, though IR data discussed below do not support this as the cause.^49^ As noted above, polymer mobility likely also plays a role, in that CO_2_ will be able to more effectively penetrate into polymer layers and agglomerates if a polymer is more mobile, thereby requiring more energy and higher temperature to expel CO_2_ from the depths of the polymer during desorption. One previous study has demonstrated that a branched PEI based sorbent had higher energies of activation for CO_2_ desorption than a linear PEI sorbent, which was attributed to the energy input required to first straighten the polymer chains of BPEI before CO_2_ can be released.^50^ Additionally, a study featuring TPD following humid CO_2_ capture suggests that the delayed desorption of CO_2_ when water was present during adsorption is due to the water-enhanced penetration of CO_2_ into the intrapore polymer film.^51^ Thus, during desorption, the elongated diffusion path for CO_2_ out of the film leads to delayed desorption peaks in the TPD profile. Similarly, in comparing the four polymer types in this current work, the higher desorption temperature of BPEI- and BPPI-impregnated SBA-15 may indicate that CO_2_ is more effective at penetrating these intraparticle polymer domains as compared to the linear species, with BPEI having the most accessible polymer nanodomains.

By repeating the TPD experiment using two additional ramp rates, we can calculate the apparent energy of CO_2_ desorption (E_d_).^52^ Figure 2B displays the energy of desorption for the four aminopolymer-impregnated SBA-15 composites at medium and high amine loadings. As noted above for the peak desorption temperatures, the higher energies of desorption for the branched species could indicate stronger bond formation due to the preferred formation of ammonium carbamate over carbamic acid (see below) and/or more effective polymer penetration and retention of CO_2_ during adsorption and desorption, respectively. Interestingly, the LPPI-impregnated SBA-15 shows a considerable increase in E_d_ from 83 to 99 kJ/mol when switching from medium to high polymer loading. Previous studies comparing linear and branched PEI-impregnated silica showed similar trends, with lower CO_2_ desorption temperatures and activation energies for LPEI than BPEI across all adsorption conditions.^36,50^ Additionally, they found that a higher polymer loading within the support correlates with a higher T_peak_ and E_d_ due to a higher diffusive barrier for CO_2_ desorption.

### *In Situ* DRIFTS

To further understand the CO_2_ reaction mechanisms at play inside the pores, *in situ* FTIR was performed in the presence of flowing 400 ppm of CO_2_ over the course of 3 h. Figure 3 shows the time evolution of the adsorbed species. While all four spectra display certain distinct bands that are common across the samples, there are some points of interest. The spectra of BPEI and BPPI show a resemblance to one another, particularly in the prominence of peaks around 1620–1630 cm^–1^ (δ_as_NH_3_^+^) and 1487–1512 cm^–1^ (νCOO^–^), previously attributed to ammonium ion and carbamate ion formation, respectively (see Table 2 for peak assignment sources). LPPI displays a shoulder around 1620 cm^–1^ (δ_as_NH_3_^+^), but LPEI largely lacks intensity at this frequency. BPPI and LPPI both display particularly sharp peaks at 1410–1420 cm^–1^, associated with carbamate C–N stretching and/or skeletal vibrations.

**Figure 3:**
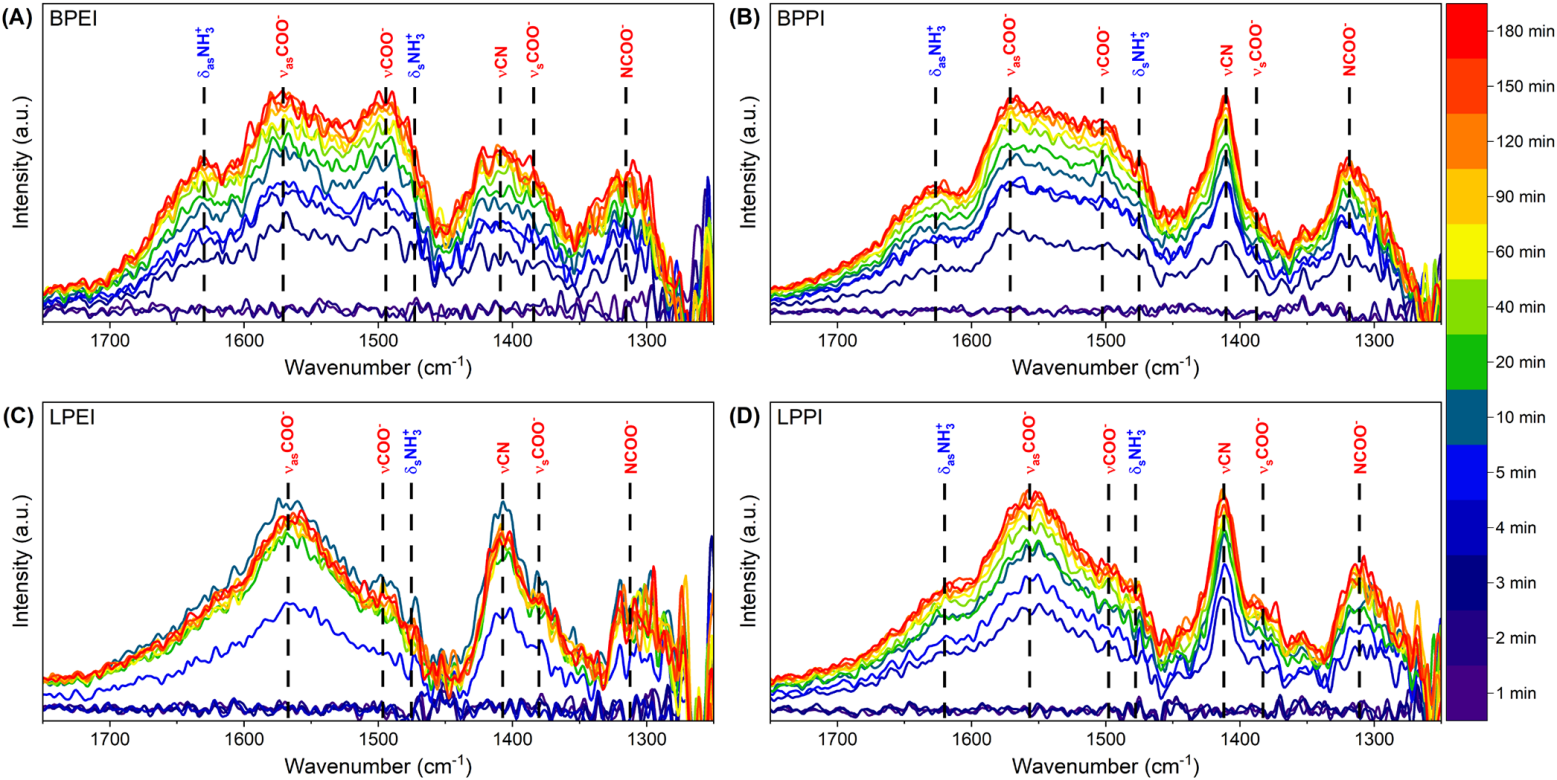
*In situ* FT-IR spectra of (A) BPEI- (B) BPPI- (C) LPEI- and (D) LPPI-impregnated SBA-15 in the presence of flowing 400 ppm of CO_2_ at 30 °C.

**Table 2:** *In Situ* IR Peak Assignments from Reaction of Aminopolymer/SBA-15 Composites in the Presence of 400 ppm of CO_2_

Wavenumber [cm^–1^]	Peak assignment	Group	Reference
1620–1630	δ_as_NH_3_^+^	Ammonium ion	^56–61^
1550–1575	ν_as_COO^–^	Carbamate ion	^53,61^
1487–1512	νCOO^–^	Carbamate ion	^56,59,61,62^
1475–1486	δ_s_NH_3_^+^	Ammonium ion	^53^
1410–1420	νCN/NCOO^–^ skeletal vibration	Carbamate ion	^54,58^ ^,^ ^61^
1385–1390	ν_s_COO^–^	Carbamate ion	^53,56,60,63^
1300–1325	NCOO^–^ skeletal vibration	Carbamate ion	^54,55,57–59^ ^,^ ^61^

Notably, none of the spectra display peaks in the 1650–1700 cm^–1^ range that have historically been attributed to the formation of carbamic acid.^53–55^ Thus, there is no support for the hypothesis that the lower desorption temperature and energy requirement observed for the linear polymers here compared to the branched polymers is associated with the formation of carbamic acid instead of carbamate. This, in addition to the fact that the TPD curves (Figure S2) display only one smooth peak, suggest that CO_2_ is only or primarily adsorbed as ammonium carbamate and that differences in desorption energy result primarily from the respective kinetic/diffusive limitations of each material.

### CO_2_ Isobars

CO_2_ isobars were obtained at constant concentrations of 400 ppm and 10% CO_2_ in nitrogen (Figure 4). In each of these experiments, the CO_2_ concentration was held constant while the temperature was changed by increments of 10 °C and held for 390 min. Exploring the adsorption performance over a wide range of temperatures enables an initial assessment regarding the type of climate zone in which a particular sorbent material might function well.^64–67^ In these experiments, the temperature was first increased to 90 °C (filled points in the figure) then decreased back to 30 °C (hollow points). It has been previously suggested that the shape of the resulting curves indicate the extent to which CO_2_ capture at a particular concentration is limited kinetically by pore packing and chain mobility.^33^ Earlier work showed that at a concentration of 10% CO_2_, the capacity of 1000 g/mol LPPI in SBA-15 first increased with increasing temperature up to 55 °C, after which it decreased during ramping up to 95 °C.^33^ Here, we replicated this experiment under 10% CO_2_ and also show the behavior under 400 ppm of CO_2_. Both the medium and highest amine loadings were tested to ascertain the impact that pore crowding may have. To compare the results across the different sample types, the data in Figure 4 are presented as ΔAE as a function of temperature, where

1 $$\Delta AE\left(T\right)=\frac{AE\left(T\right)-AE\left(30{}^{\circ}C\right)}{AE\left(30{}^{\circ}C\right)}\times 100$$

and AE­(T) is the amine efficiency at each temperature, with AE­(30 °C) the amine efficiency at 30 °C.

**Figure 4:**
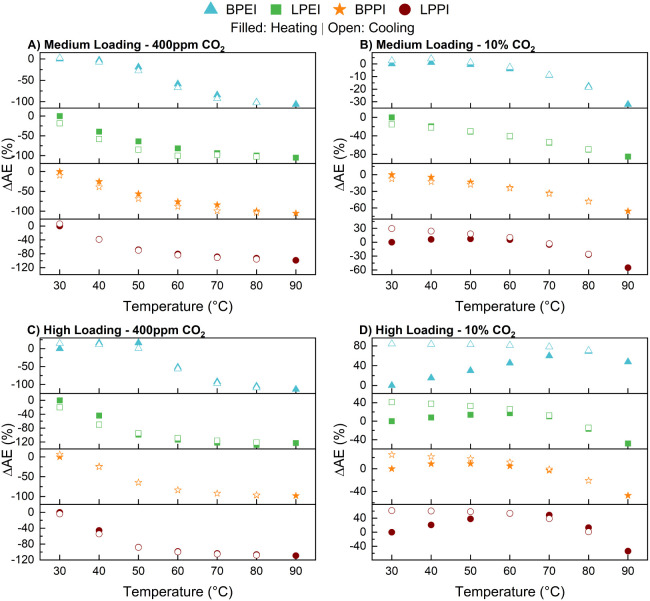
Change in amine efficiency with a change in adsorption temperature under a constant stream of CO_2_. ΔAE represents the percent change in amine efficiency compared to its value at 30 °C. The rows (A,B and C,D) represent the medium and high amine loading levels, respectively. The columns (A,C and B,D) represent CO_2_ concentrations of 400 ppm and 10%, respectively. Closed and open symbols represent data collected while ramping up and ramping down, respectively, during these isobar measurements.

As can be seen at the medium polymer loading and under 400 ppm of CO_2_ (Figure 4A), all samples only experience a decrease in capacity with an increase in temperature. This suggests that regardless of the polymer architecture, the adsorption thermodynamics are the limiting factor, as opposed to the CO_2_ diffusion rate, such that increased amine mobility has no effect on CO_2_ adsorption under DAC conditions. In the high amine loading scenario at 400 ppm (Figure 4C), only the BPEI sample displays a slight enhancement with the increase in temperature, peaking at 50 °C with a 16% improvement before decreasing. Since the corresponding LPPI and BPPI sorbents had similar and even higher levels of pore filling and displayed no improvement with the temperature increase, this result indicates that BPEI has superior mobility within the pores among the polymers studied here.

Under 10% CO_2_, the picture is quite different. At this concentration the rate of adsorption begins to outpace the rate of CO_2_ diffusion such that diffusive limitations become a contributing factor. At the highest loadings, all materials see a jump in uptake at higher temperatures (Figure 4D). Branched PEI improves the most during heating (+69%) and peaks at the highest temperature across all experiments (80 °C), while BPPI improves the least (+9%) and peaks at only 50 °C. The opposite trend is true of the linear species. Though both linear polymers peak at 60 °C during heating, LPPI sees a 53% increase while LPEI only achieves a 17% increase. Interestingly, and as shown previously by Pang et al., all samples see a steady increase in capacity upon cooling, displaying maximums at 30 °C.^33^ It was formerly suggested that this is due to the opportunity for CO_2_ to penetrate bulk polymer layers at elevated temperatures due to higher CO_2_ diffusivity and increased polymer mobility, reacting with amine sites that were previously inaccessible at lower temperatures.

At the medium loading and under 10% CO_2_, only BPEI and LPPI display capacity increases with heating, though to a lesser extent than at the high loading (Figure 4B). The capacities of linear PEI and branched PPI only decrease with heating under these conditions. The difference seen across these two loading levels can be attributed to the pore packing. At the highest loading, the pores of the composites, with the exception of LPEI, are close to being completely filled. In this case, as has been previously identified for BPEI/SBA-15 systems, the walls of the pores are completely covered and plug-like aggregates of polymer form, choking axial diffusion of CO_2_ deeper into the pores.^68^ Therefore, these composites would benefit the most from the diffusive benefits brought about at higher temperatures.

It can also be the case that the significantly different trends at the two CO_2_ concentrations are due to the degree of polymer chain cross-linking during the capture process.^69,70^ Two amine sites are required for ammonium carbamate formation and whether done inter- or intramolecularly, these pairs form a cross-link that increases the rigidity of the polymer. Since sorption capacity is dependent on the partial pressure of CO_2_, at 10% there is greater formation of ammonium carbamate pairs and a higher extent of cross-linking. Thus, a temperature increase is even more important to overcome this more significant barrier at 10% CO_2_ conditions, beyond the intrinsic barrier of polymer agglomeration.

### CO_2_ Sorption/Desorption Cycling

To explore the long-term stability implications of the four different composite types, 25 temperature-swing adsorption–desorption cycles were performed (Figure 5). Following a 3-h initial activation at 110 °C under nitrogen, adsorption occurred at 30 °C for 1 h under 400 ppm of CO_2_, followed by a 10 min desorption step at 110 °C in N_2_. Figure 5A and C show the amine efficiency at the end of each adsorption step as a function of the cycle number. All four aminopolymers at both loadings demonstrate decent stability over the TSA cycles. Only BPPI experiences a noteworthy decline in amine efficiency between the first and 25th cycles, by 10% and 11% for the medium and high loading, respectively. Figure 5B and D show the changes in the sample mass at the beginning of each adsorption stage over the course of the experiment. Overall, the decay seen is minimal, with a maximum change of about −0.8% for the high loading BPPI-impregnated SBA-15. At both the high and medium loadings, the linear polymers retain their mass more effectively than the branched polymers. Sample mass decay, particularly to the low degree seen with the linear polymers, could be due to the presence of tough-to-remove water that eventually escapes over repeated dry gas thermal cycling, with the initial 3-h activation period provided at the beginning of the experiment insufficient to remove all water in the first step. As has been previously observed, BPEI is more hydrophilic than LPEI, adsorbing up to 32% more water, which is partly attributed to the different bulk phase of BPEI (viscous liquid) and LPEI (solid).^38^ Additionally, the cyclic mass loss could also be due to the presence of low molecular weight oligomeric amines in the branched polymer composites that volatilize during the high temperature desorption period. This could then explain why BPPI suffers from a slight decline in amine efficiency during cycling. Polydisperse, oligomeric amines inherently contain a higher fraction of primary amines than polyamines with a higher degree of polymerization. These primary amines can play an outsized role at low CO_2_ concentrations and particularly with a short adsorption period of only 1 h.

**Figure 5:**
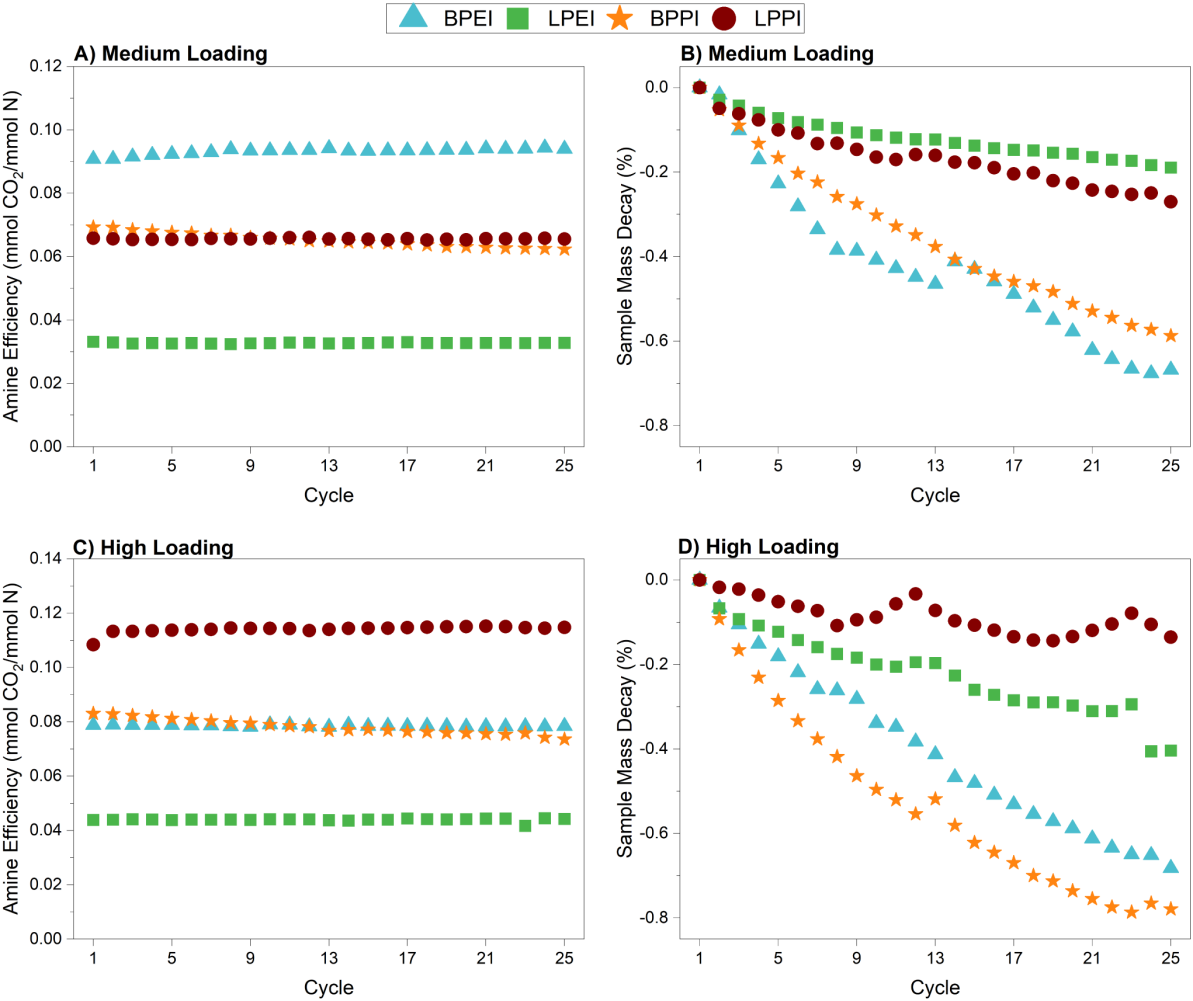
CO_2_ adsorption–desorption performance over 25 cycles with temperature swing regeneration. A&C) Amine efficiency as a function of adsorption cycle. Mass change during adsorption was determined using the mass at the beginning of each cycle. B&D) Change in starting sample mass as a function of adsorption cycle. Adsorption performed at 30 °C under 400 ppm of CO_2_ for 1 h. Desorption performed under N_2_ at 110 °C for 10 min.

## Conclusions

Composite sorbents of PEI and PPI, in both branched and linear architectures, physically impregnated into SBA-15 were tested for their application in direct air capture. Linear and branched PPI as well as linear PEI were synthesized to ensure similar polymer molecular weights across all varieties. The adsorption, desorption and cyclic behavior and performance were investigated under dry conditions. Additionally, a short survey of capacity at elevated temperatures was performed, and *in situ* IR measurements were collected to identify the sorbed species.

The above comparison revealed several insights into how polymer architecture influences CO_2_ capture performance under DAC-relevant conditions. LPPI-impregnated SBA-15 sorbents achieved the highest amine efficiency across all loading levels, reaching up to 0.14 mmol CO_2_/mmol N, slightly surpassing the BPEI composite. Meanwhile, LPEI exhibited the lowest capacities and amine efficiencies, consistent with previous experimentation, possibly due to its limited polymer mobility and tighter packing within pores. Temperature-programmed desorption studies highlighted that the branched polymers exhibited higher CO_2_ desorption energies (102–111 kJ/mol) compared to their linear analogues, suggesting stronger polymer–CO_2_ interactions or more effective CO_2_ penetration into polymer domains. *In situ* DRIFTS confirmed that all sorbents captured CO_2_ primarily as ammonium carbamate. Additionally, isobaric CO_2_ uptake tests demonstrated that BPEI maintained better performance under elevated temperatures and concentrations, likely benefiting from enhanced polymer mobility and pore accessibility.

Several limitations exist in extrapolating the results from this model study to practical systems. SBA-15 is not a commercially available support, so future studies should focus on transitioning the aminopolymers into industry-grade alumina or silica often applied in CO_2_ capture. Furthermore, though the short-term cyclic studies presented above show no immediate concern of material loss, these materials need to be shown to be resilient across thousands of adsorption/desorption cycles under realistic desorption conditions.^22^ Finally, PPI can only be accepted as a possible alternative after its performance is analyzed under more practical conditions, particularly involving humidified gas streams.

## Supplementary Material



## Notes

CO_2_ isotherms (400 ppm) and uptake data; TPD curves; energy of desorption determination plots; combustion TGA plots; MALDI-TOF results for synthesized branched PPI;^1^H NMR for synthesized linear PPI and PEI; nitrogen physisorption isotherm for SBA-15; amine-normalized pore fill; XPS; powder XRD of LPPI; monomer and polymer molecular structures (PDF)
