# Supplementary material for: Role of Polymer Architecture in CO2 Capture from Air Using Supported Poly(alkylenimine)s: Linear vs Branched Polymers
Source: ACS Appl Polym Mater. 2025 Nov 17;7(22):15671–81. doi: 10.1021/acsapm.5c03465 (PMC12670363; doi:10.1021/acsapm.5c03465)
Supplement: Supplementary file 1 [file ap5c03465_si_001.pdf]

Supporting Information

Role of Polymer Architecture in CO_2 Capture from Air using Supported Poly(alkylenimine)s: Linear vs. Branched Polymers

Jacob Hoffman, Laura Proaño, Christopher W. Jones

School of Chemical & Biomolecular Engineering, Georgia Institute of Technology, 311 Ferst Dr., NW, Atlanta, GA, 30332, USA

Email: cjones@chbe.gatech.edu

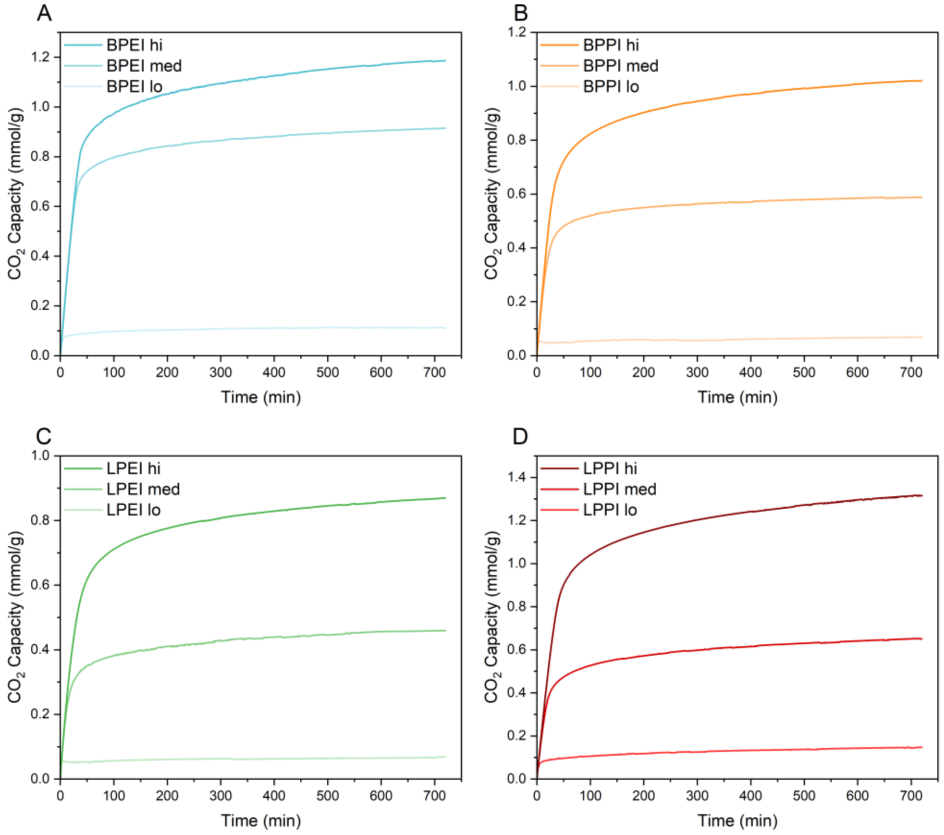

Figure S1. CO_2 uptake curves of (A) BPEI-, (B) BPPI-, (C) LPEI- and (D) LPPI-impregnated SBA-15 composites. Adsorption performed for 12 hours at 30°C under 400pm CO_2/N_2 balance following 1 hour activation at 110°C. Performed on TA Instruments TGA 550.

Table S1. CO_2 capacity (mmol CO_2 /g sorbent) and amine efficiency (mmol CO_2/mmol N) following 12 hour adsorption experiments under 400ppm CO_2 on TA Instruments TGA 550.

	CO_2 Capacity [mmol CO_2/g]	Amine Efficiency [mmol CO_2/mmol N]
BPEI low	0.10	0.02
BPEI mid	0.82	0.10
BPEI high	1.14	0.11
LPEI low	0.07	0.02
LPEI mid	0.46	0.07
l-PEI high	0.87	0.09
BPPI low	0.07	0.02
BPPI mid	0.57	0.08
BPPI high	1.00	0.12
LPPI low	0.15	0.04
LPPI mid	0.65	0.10
LPPI high	1.32	0.14

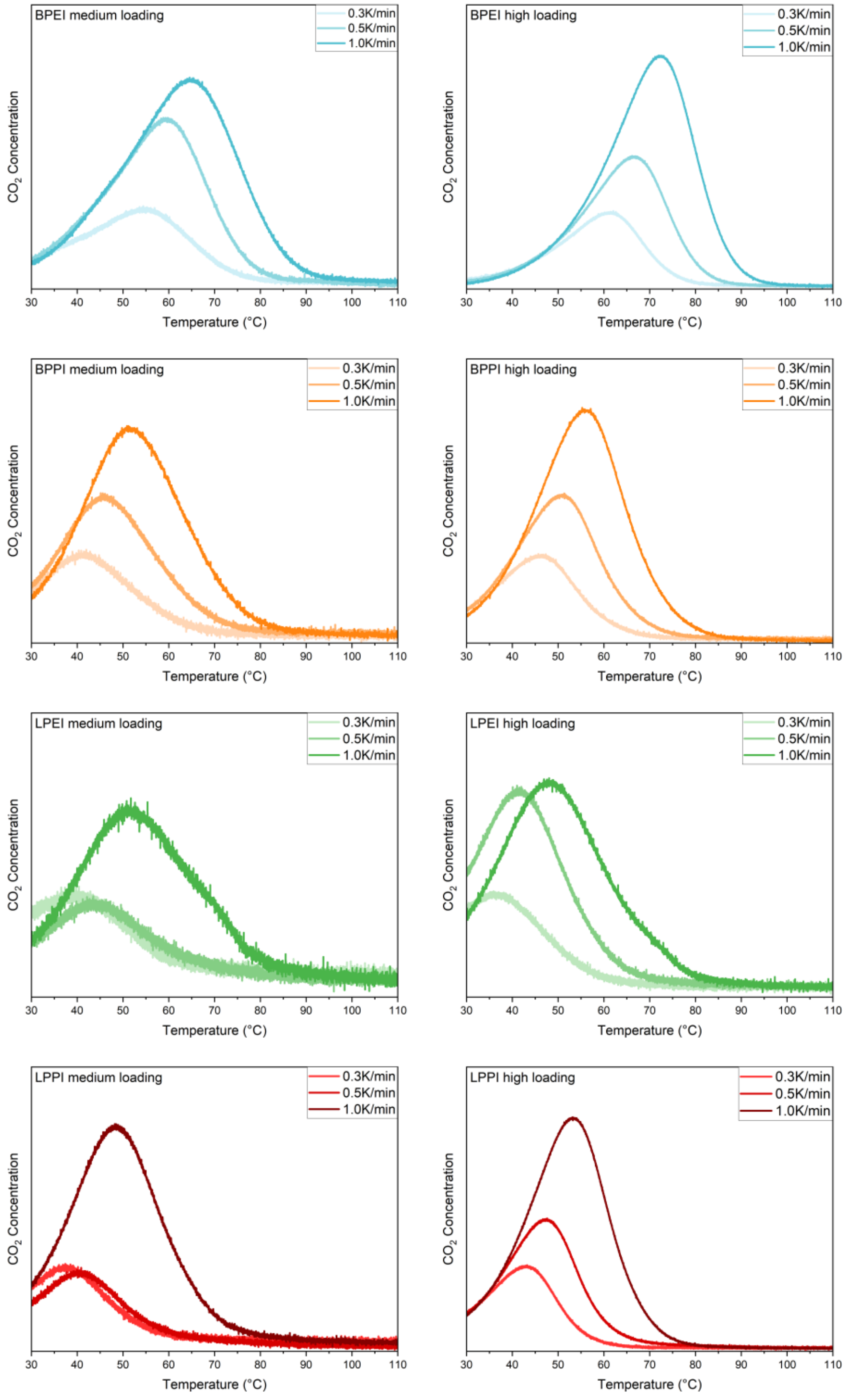

Figure S2. Temperature programmed desorption curves monitoring the CO_2 concentration of the outlet during desorption at a fixed ramp rate. Three ramp rates were employed (0.3, 0.5 and 1.0 °C/min) following respective 12-hour adsorption phases under 400 ppm CO_2 at 30 °C. Performed on TA Instruments TGA 550. CO_2 concentration measured by a LI-COR LI-830 Gas Analyzer.

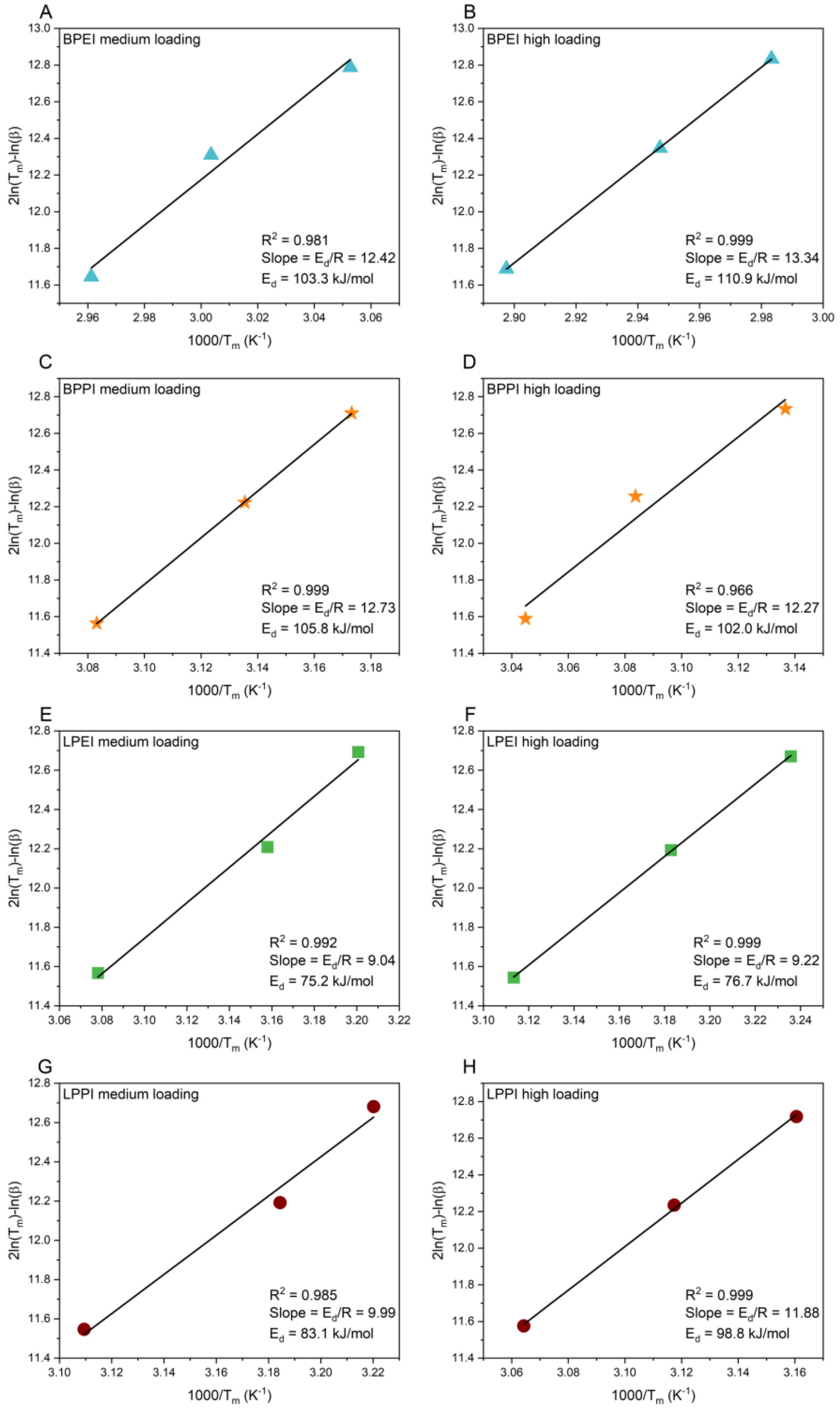

Figure S3. Determination of the energy of CO_2 desorption based on microkinetic analysis for (A/C/E/G) medium and (B/D/F/H) high aminopolymer-loaded SBA-15 following dry 400 ppm CO_2 adsorption at 30 °C. Method taken from Cvetanović et al.[1]

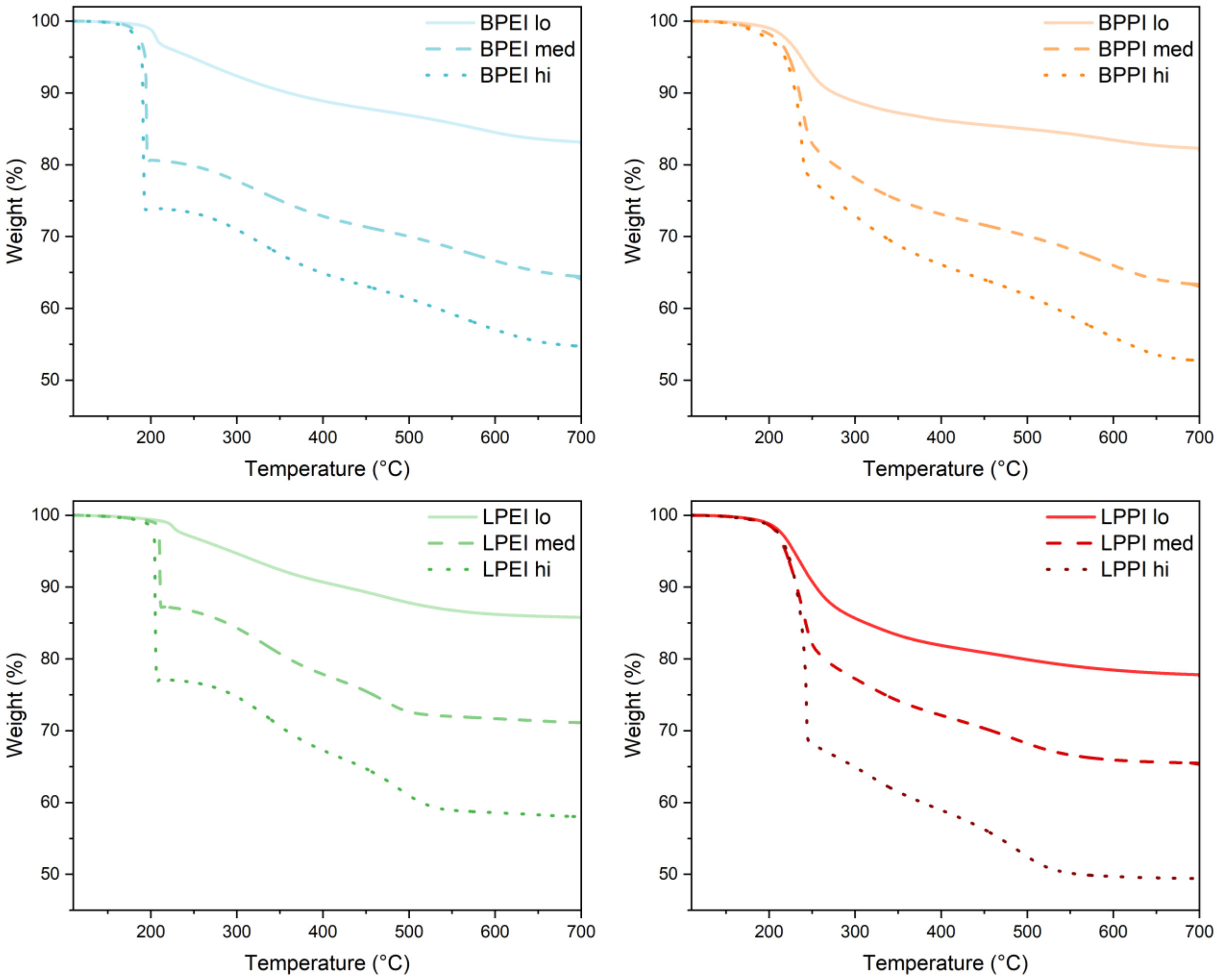

Figure S4. Combustion TGA profiles for calculation of organic and amine loadings of the composites. Performed on TA Instruments TGA 550.

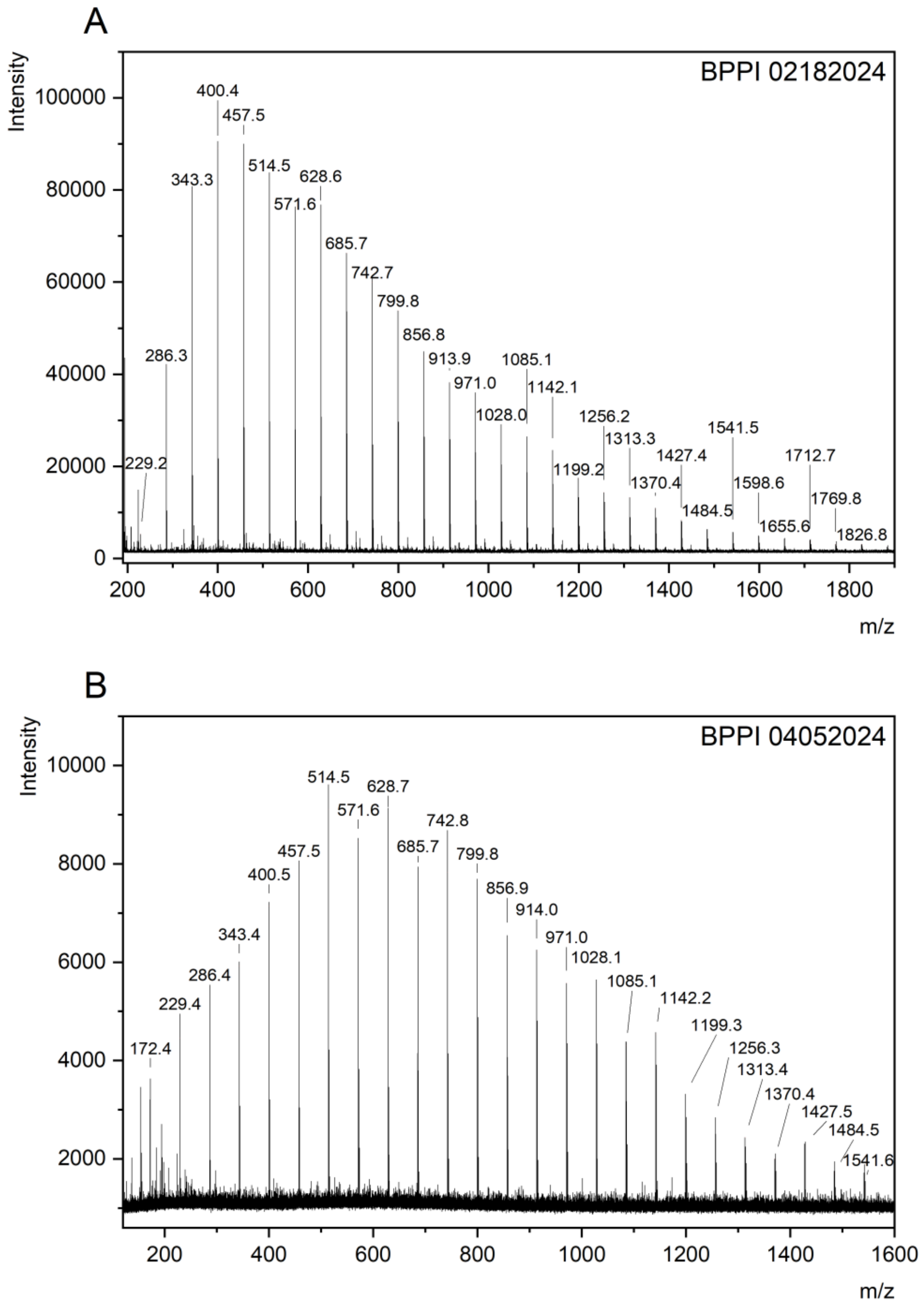

Figure S5. MALDI-TOF spectra for two batches of synthesized branched poly(propylenimine) used in this study. (A) M_n = 730 g/mol; M_w = 890 g/mol; Đ = 1.22. (B) M_n = 757 g/mol; M_w = 904 g/mol; Đ = 1.19

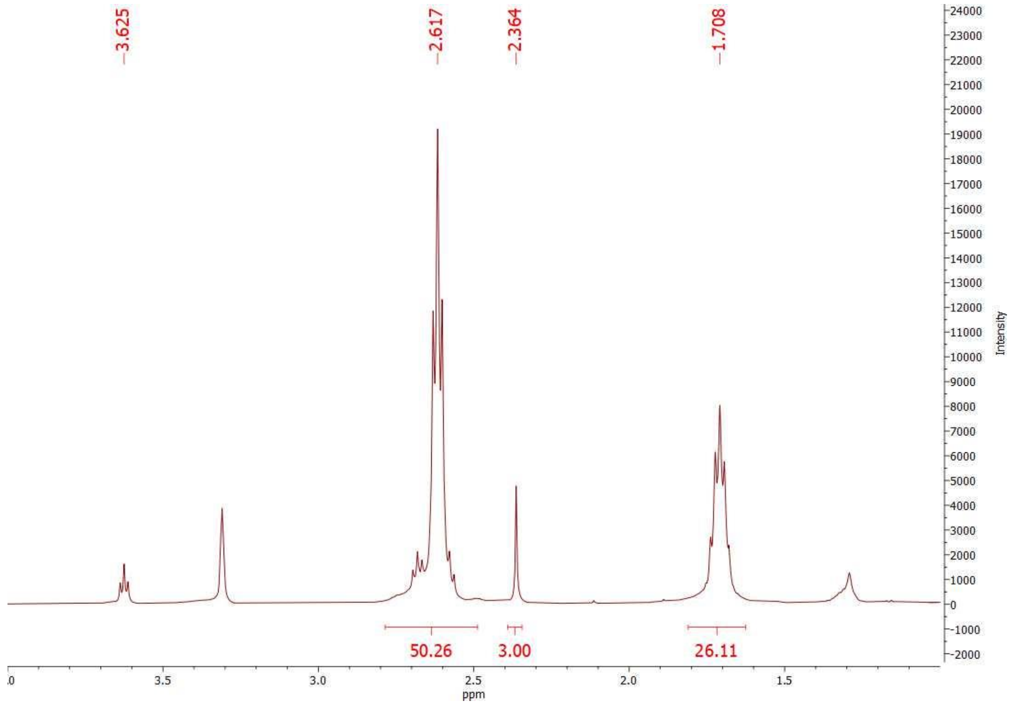

Figure S6. ^{1}H NMR (500 MHz; CD_3OD) of synthesized linear poly(propylenimine). Peak assignments and molecular weight determination methods taken from Pang et al.[2]

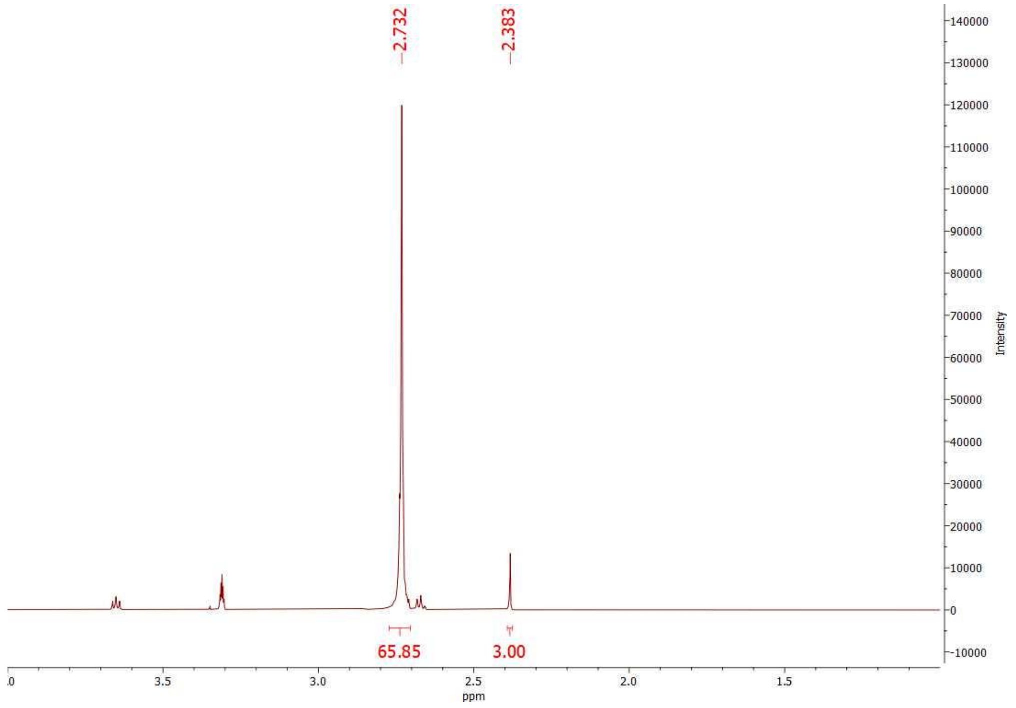

Figure S7. ^{1}H NMR (500 MHz; CD_3OD) of synthesized linear poly(propylenimine).

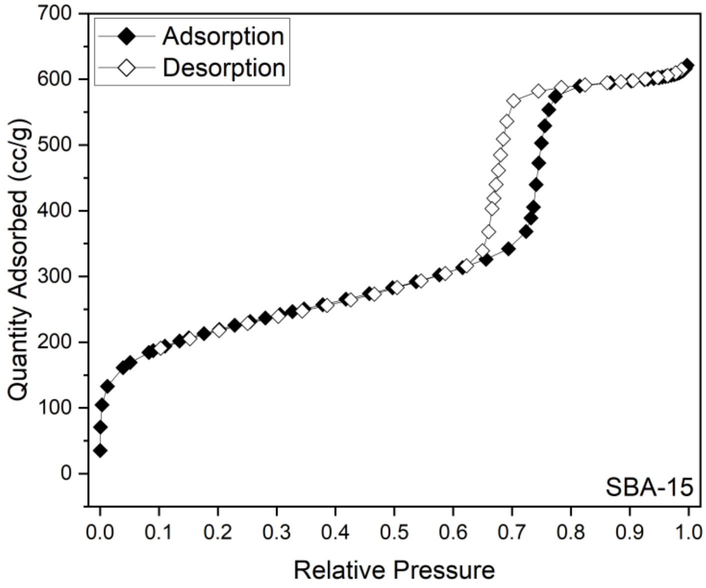

Figure S8. Nitrogen physisorption isotherms at 77 K of the bare SBA-15 silica support.

Table S2. Amine-normalized pore filling of the aminopolymer-impregnated composites. Final column calculated by dividing the third column by the second.

Sample	Amine Loading [mmol N/g support]	Pore Fill [$PV_{composite}/PV_{SBA-15}$]	Amine-normalized pore fill
BPEI low	4.9	30	6.1
BPEI mid	13	72	5.5
BPEI high	19.5	91	4.7
LPEI low	4.1	23	5.6
LPEI mid	9.8	48	4.9
LPEI high	17.1	68	4.0
BPPI low	3.9	36	9.2
BPPI mid	10.3	73	7.1
BPPI high	15.9	95	6.0
LPPI low	4.9	27	5.5
LPPI mid	9.6	60	6.3
LPPI high	18.7	97	5.2

Table S3. Chemical composition of selected composite samples via XPS and combustion TGA.

		Blank SBA-15	LPEI low	LPEI high	BPEI high
XPS	O %	63.8	56.4	41.0	32.6
	N %	0.3	3.6	11.3	16.4
	C %	2.2	8.5	24.9	32.0
	Si %	33.6	31.5	22.7	19.1
	C/Si	0.07	0.3	1.1	1.7
	N/Si	0.01	0.1	0.5	0.9
	C/N	6.4	2.4	2.2	1.95
TGA	N%	--	4.9	13.9	14.7

The LPEI-based samples, hypothesized to have a disproportionately high concentration of deposited polymer on the support external surface because of their low pore fill percentage, display lower N/Si ratios via XPS than the sample with the highest BPEI loading. This suggests that the LPEI is not disproportionately deposited on the external surface. Additionally, the N%, as measured by XPS, is lower than the N% calculated via TGA combustion data, which captures the general weight percentage of combustible material (polymer) in the samples. This similarly suggests that the polymer is sufficiently deposited within the internal pore structure of the support.

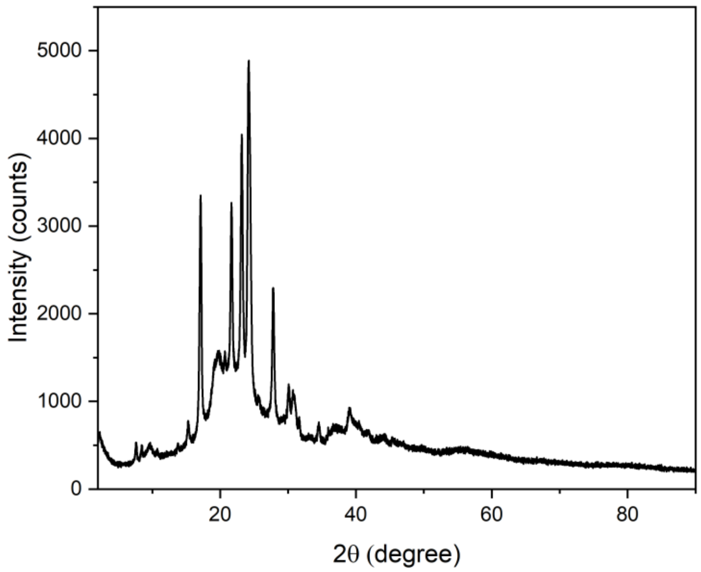

Figure S9. Powder XRD of bulk linear poly(propylenimine). Performed on Rigaku Miniflex.

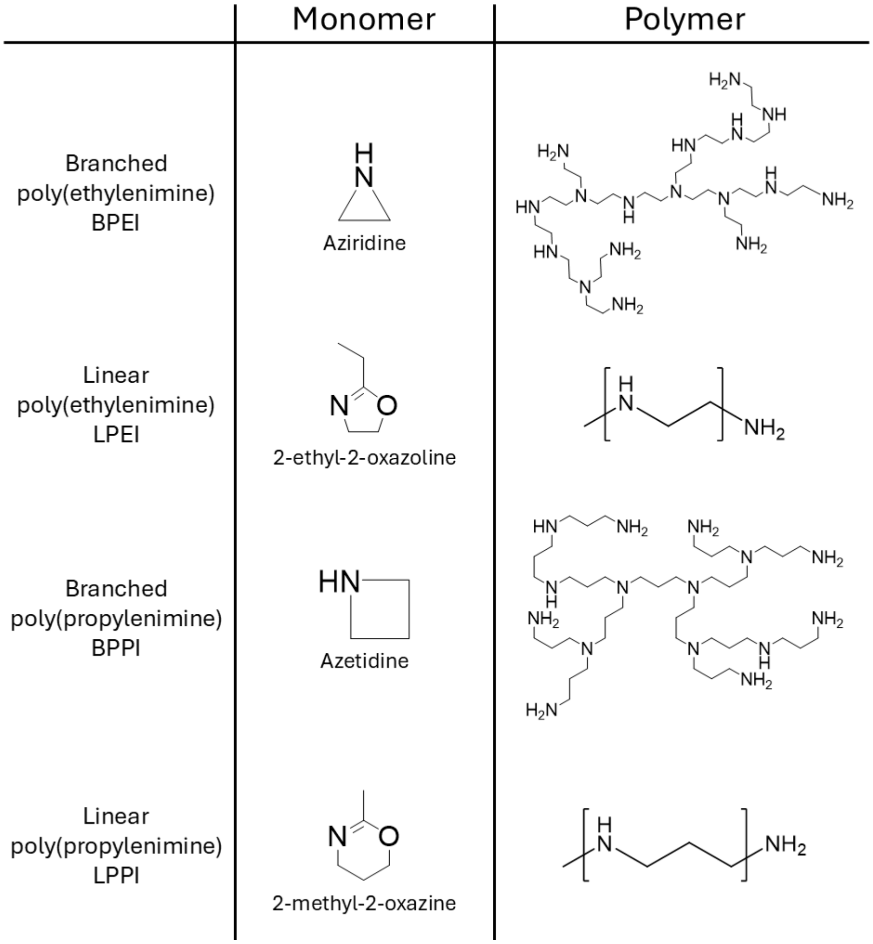

Figure S10. Molecular structures of monomers and polymers used in the study

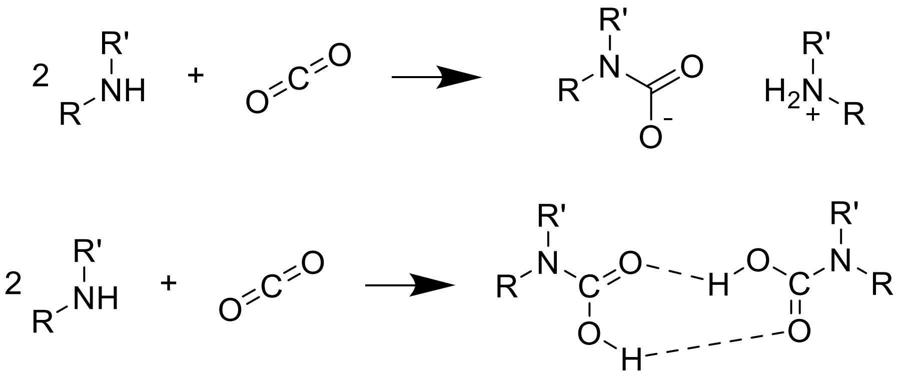

Figure S11. Formation of ammonium carbamate (top) and carbamic acid (bottom) via the reaction of two amine sites with carbon dioxide. R' = H for primary amine, = $-CH_2-$ for secondary amine

References

(1) Cvetanović, R. J.; Amenomiya, Y. Application of a Temperature-Programmed Desorption Technique to Catalyst Studies. *Advances in Catalysis* **1967**, *17*, 103–149. https://doi.org/10.1016/S0360-0564(08)60686-0.

(2) Pang, S. H.; Lively, R. P.; Jones, C. W. Oxidatively-Stable Linear Poly(Propylenimine)-Containing Adsorbents for CO_2 Capture from Ultradilute Streams. *ChemSusChem* **2018**, *11* (15), 2628–2637. https://doi.org/10.1002/cssc.201800438.
